# Commentary: High-metastatic melanoma cells promote the metastatic capability of low-metastatic melanoma cells *via* exosomal transfer of miR-411-5p

**DOI:** 10.3389/fonc.2022.957035

**Published:** 2022-08-22

**Authors:** Tianhang Li, Hongqian Guo, Rong Yang

**Affiliations:** Department of Urology, Affiliated Nanjing Drum Tower Hospital, Medical School, Nanjing University, Nanjing, China

**Keywords:** tumor metastasis, exosome, intercellular communication, tumor evolution, miRNA, immune escape

Exosome-encapsulated bioactive molecules are known to play a pivotal role in modulating a variety of malignant biological behaviors, especially metastasis. Due to its high content fidelity, long-distance transportation capacity, and abundant material loading capabilities, the exosome has been verified as a good trainer for the formation of a premetastatic niche with a range of protumoral features. Recently, a study by Chen et al. demonstrated a unique function exerted by melanoma cancer cells to enhance metastatic capability through exosomal transfer from high-metastatic cells to low-metastatic cells (1). This is a very interesting article, which enriches and expands the functions and mechanisms of exosomes in cancer. Our team previously published a hypothesis article in the journal (2), whose topic has a tight connection with this research, and we are very interested in this issue. Of note, we would like to propose and highlight some points based on this article and our experience.

We would like to start by asking: What does “high-metastatic tumor cells” mean? Even now, there may not be a definite answer to the question. Traditionally, cancer cells are viewed as undergoing an everlasting Darwinian evolutionary selection process during metastasis, which determined that there would be multiple competing subpopulations within the primary tumor lesion. Of note, polyclonal seeding has been identified in both mouse models and humans, and this provided high incidences of metastasis-to-metastasis seeding, which further added to the complexity of the metastasis process (3, 4). More importantly, recent studies have indicated that the so-called high-metastatic capacities could be acquired exogenously through epigenetic reprogramming modulation within the metastatic microenvironment (5), which suggested that the high-metastatic phenotypes of a single cancer cell, a single subclone, or polyclones may not be innate and could be acquired through the crosstalk with the environment. Returning to this article, the authors obtained the high-metastatic cell line and the low-metastatic cell line through *in-vivo* selection and passage, which is an ingenious approach originally established by Sun et al. (6). Moreover, the authors verified the enhancement of the metastatic capability through *in-vitro* experiments, which mainly focused on the migration and proliferation phenotypes. Although the displayed variation is significant, we thought that the *in-vivo* verification may be a more solid proof. Moreover, it is unclear whether the MDA-MB-435 breast cancer cell line that Sun et al. used or the M14 melanoma cancer cell line that this article utilized tends to firstly metastasize to the lymph nodes rather than the lung. Combined with the results of previous studies, which verified that those metastatic cancer cells which colonized further organs are derived from the second metastatic niche rather than the primary mass, we may overlook the possible epigenetic reprogramming that happened during the first colonization. This may limit the future clinical applicable translational value of this research.

Recently, Reticker-Flynn et al. (7) have also utilized a similar *in-vivo* repeating selection approach to generate high-metastatic melanoma cell strains. Interestingly, they gave an explanation for the enhancement of metastatic capabilities that the immune escape phenotypes, which are mainly represented by MHC-I and programmed death ligand 1 (PD-L1) expression, were amplified by the IFN-γ signaling pathway. In addition, Zhang et al. also found that in breast cancer, the upregulated metastatic capabilities of further metastatic cancer cells were conferred by the bone microenvironment through stemness enhancement, which promotes their secondary colonization to distant organs (5). These reminded us that the phenotypes of the metastatic capacities of cancer cells are not only just the upregulation of migration and proliferation abilities but also the remodeling of multiple biological characteristics determined by the distinct features of the microenvironment of the first metastatic niche.

The authors performed a solid validation on the exosome-mediated transfer of miRNA-411-5p and the subsequent effect of invigorating cancer cells with higher migration and proliferation capabilities, which is an intriguing functioning mechanism. Here, we want to highlight a few points related to this issue. Firstly, the “cargoes” that the exosomes transferred may not only be capsuled within them but also be carried on the surface of their membrane, which may allow the cell-to-cell long-distance exchange of membrane proteins. Previously, the exosome-loaded PD-L1 was found capable of passing through CD8^+^ T cells and subsequently suppressed their anticancer immunity (8). Therefore, future studies may focus on not only the exosome-capsuled molecules but also the exosome membrane proteins such as some crucial immune checkpoint proteins like CD47, MHC-I, and PD-L1, especially under the premise that some high-metastatic cancer cells possessed a higher expression of such immune checkpoint proteins. In addition, we suppose that the interactions between the high-metastatic cancer cells and the low-metastatic cancer cells may be categorized into two main modes. The first mode may be categorized according to what was demonstrated by Chen et al. in this article—that the exosomes are transferred from the donor high-metastatic cancer cells to the low-metastatic receivers. However, we suspect that this process would mainly be underway between two independent subclones located at a distance, which is mainly due to the advantageous capabilities of exosomes to transfer bioactive modulatory molecules with great stability and the good absorption efficiency of the receiver cells. Meanwhile, for the individual tumor mass, we thought that the exosome-mediated cell-to-cell communication may not necessarily be the predominant way, because the direct secretion and ingestion of bioactive molecules, such as cytokines or chemokines, are sufficient for the short-distance intracellular communication. Reticker-Flynn et al. verified that the high-metastatic lymph node-colonized cancer cells could promote T-cell differentiation into regulatory T cells through TGF-β secretion (7). Although this process is conducted between cancer cells and immune cells, we may naturally hypothesize that similar activities could also happen between high-metastatic cancer subclones and low-metastatic subclones within the primary tumor mass. However, this needs to be verified in future investigations.

In addition to the exosome-exerted functions *via* its loaded bioactive molecules, the mechanism by which the exosomes act is another important target for carcinogenesis inhibition and drug discovery. This process could be mainly divided into two subunits: the production of exosomes from the donor cells and the uptake of exosomes by the recipient cells.

As for the production or secretion of exosomes, we may need to first review the formation process of exosomes. At the initiating step of endocytosis, the endosomes firstly originated from the internalization of the cell membrane. Then, the endosome membrane would invaginate and form abundant small vesicles inside the endosome, which thereby formed the multivesicular body (MVB). Finally, the MVBs would get fused with the cell membrane and secrete the internal small vesicles into the extracellular space, which subsequently became the exosomes we discussed here (9, 10). Obviously, the production of exosomes determined to a large extent the driving force of the exosome function in the tumor microenvironment. Previous studies found that Rab27 and its downstream effector molecules predominately mediate the production of exosomes (11). Also, the secretion of exosomes could be further promoted by the p53/TSAP6 axis (12). Based on the regulatory roles played by these molecules on the production of exosomes, a number of studies have been performed to investigate the anticancer efficacy of the blockade of exosome production. Guo et al. found that inhibition of Rab27a could significantly suppress the progression of melanoma through disruption of the biogenesis of a distinct proinvasive exosome population (13). Interestingly, Wang et al. illustrated that a long non-coding RNA (lncRNA) could inhibit the production of exosomes *via* binding with Rab5 mRNA and the subsequent decrease of its stability, which further suppressed the development of colon cancer (14). In summary, the inhibition of the production of oncogenic exosomes is a promising target for anticancer drug discovery in the future.

Meanwhile, the uptake of the exosomes by the recipient cells is another important regulatory pathway, of which the process could be mainly categorized into three steps. Firstly, a direct interaction would occur between the exosome membrane proteins and the signaling receptors of the recipient cell membrane (15). Secondly, following the interaction, the membrane of the exosome would fuse with the cell membrane of the recipient cells and then secrete the internal molecules into the cytosol of the recipient cells (16). After the membrane fusion, the exosome would get internalized by the recipient cells and further undergo degradation or transcytosis (17). Undoubtedly, the uptake of the exosomes by the recipient cells and the involved regulatory mediators are also crucial participators in the biological functioning process of exosomes. The structural damage of the exosome lipid rafts was found to inhibit the absorption of exosomes, which subsequently alleviated the progression of breast cancer (18). Furthermore, another study determined that the administration of protease K could effectively suppress the uptake of exosomes in breast cancer (19). Although the exact mechanism of exosome uptake is still unclear, more discovered targets would for sure provide us with more strategies to regulate the exosome function and, thus, achieve better anticancer effects.

In addition, we would like to point out that according to the latest recommendations and study requests of the International Society for Extracellular Vesicles (ISEV), the collective term “small extracellular vesicles” should be used instead of “exosomes.” The main reason is that the generation pathways and distinct surface biomarkers of exosomes are heterogeneous. Thus, in the absence of the small extracellular vesicle characterization or data support for the type of small extracellular vesicle studied, we should use the term “small extracellular vesicles” instead of “exosomes” or other specific names (20).

To summarize, the authors performed an interesting and thought-provoking research on the mechanism of cancer metastasis. The intercellular communication *via* exosome transfer between different tumor subclones would for sure open a new door for oncology research.

## Author contributions

RY and HG organized and summarized the scientific ideas and participated in the discussion. TL wrote the manuscript and participated in the discussion. All authors contributed to the article and approved the submitted version.

## Funding

This work was supported by the National Natural Science Foundation of China (82172691 and 81772710) and Nanjing Science and Technology Development Key Project (YKK19011).

## Conflict of interest

The authors declare that the research was conducted in the absence of any commercial or financial relationships that could be construed as a potential conflict of interest.

## Publisher’s note

All claims expressed in this article are solely those of the authors and do not necessarily represent those of their affiliated organizations, or those of the publisher, the editors and the reviewers. Any product that may be evaluated in this article, or claim that may be made by its manufacturer, is not guaranteed or endorsed by the publisher.
